# Commentary: Cerebellar atrophy and its contribution to cognition in frontotemporal dementias

**DOI:** 10.3389/fnagi.2018.00300

**Published:** 2018-10-01

**Authors:** Agustín Ibáñez, Adolfo M. García

**Affiliations:** ^1^Institute of Cognitive and Translational Neuroscience (INCYT), INECO Foundation, Favaloro University, Buenos Aires, Argentina; ^2^National Scientific and Technical Research Council (CONICET), Buenos Aires, Argentina; ^3^Center for Social and Cognitive Neuroscience, School of Psychology, Universidad Adolfo Ibanez, Santiago, Chile; ^4^Department of Psychology, Universidad Autónoma del Caribe, Barranquilla, Colombia; ^5^Centre of Excellence in Cognition and its Disorders, Australian Research Council, Sydney, NSW, Australia; ^6^Facultad de Educación, Universidad Nacional de Cuyo, Mendoza, Argentina

**Keywords:** cerebellum, frontotemperal lobar degeneration, cognition, imaging, language, social cognition

## The embodied little brain: from neurocognition to neurodegeneration

Chen et al. (2018) provide unprecedented evidence of syndrome-specific changes in cerebellar gray matter integrity (mainly in lobules VI, Crus I and Crus II) across three frontotemporal dementia (FTD) subtypes, alongside specific associations with attentional, visuospatial, mnesic, and language-motor deficits. Moreover, results survived covariation with each group's distinctive atrophy pattern. These outcomes illuminate the critical role of the cerebellum in non-motor processes, while highlighting the relevance of distributed network approaches to cognitive (dys)function.

Although the cerebellum has been implicated in higher-order domains (Roca et al., 2013; García et al., 2017; Sokolov et al., 2017), including executive functions, language, interoception, and social cognition, these results may prove surprising to many clinical neuroscientists. Indeed, the cerebellum remains notably underexplored within neurocognitive assessments of dementia, where it is still largely conceived as a specifically motoric region and is thus often excluded from imaging analyses seeking to map brain-behavior associations. Similarly, except for research on ataxia, systematic assessments of regional, and network-level alterations involving the cerebellum are wanting in the field. This counterproductive neglect, we believe, stems from a dissociation between dementia studies and current neurocognitive theories (Ibáñez and García, 2018).

Fertile ground could be gained by anchoring neurodegeneration research on the embodied cognition approach, which has revealed multidimensional links between action-related circuits and higher-order functions. The cerebellum, as a core hub in these cortical-subcortical networks, would play an important, enactive role in several cognitive processes. While lesion and agenesia studies suggest that this role may not be causal, cerebellar circuits have been directly implicated in embodied domains (Koziol et al., 2012; Birba et al., 2017; García et al., 2017; Cervetto et al., 2018). Beyond the field's traditional focus on canonical atrophy patterns and selected cognitive skills, emerging evidence suggests that diffuse neurocognitive dysfunctions are partially overlapped across dementias. The profuse interconnectedness, functional richness, and transdiagnostic vulnerability of the cerebellum render it a key target to examine embodied cognitive deficits in FTD and other conditions.

Accordingly, embodied theories of the cerebellum could become critical tools to foster relevant translational developments. First, they underscore the need to systematically report cerebellar involvement in diverse neurocognitive deficits. Also, they provide a profitable platform to track intercognitive phenomena—enactive synergies among varied psychobiological (dys)functions—from a network-based perspective (Koziol et al., 2012; Ibanez et al., 2014, 2018; García and Ibáñez, 2016; Birba et al., 2017; García et al., 2017; Cervetto et al., 2018; Ibáñez and García, 2018). Moreover, they promote a reinterpretation of symptoms from an action-grounded neurocognitive rationale (Krakauer et al., 2017). These milestones could have direct clinical implications, as the lack of proper theoretical frameworks can lead to neglecting, downplaying, or delaying the report of cerebellar disturbances across pathologies. We call for novel studies integrating embodied, intercognitive, network-based conceptualizations of the cerebellum to foster translational breakthroughs in dementia research.

## Author contributions

AI: conception and design. AI and AG: drafting the manuscript.

### Conflict of interest statement

The authors declare that the research was conducted in the absence of any commercial or financial relationships that could be construed as a potential conflict of interest.

## References

[B1] Birba A. García-Cordero I. Kozono G. Legaz A. Ibáñez A A. Sedeño L. Birba L.. (2017). Losing ground: Frontostriatal atrophy disrupts language embodiment in Parkinson's and Huntington's disease. Neurosci. Biobehav. Rev. 80, 673–687. 10.1016/j.neubiorev.2017.07.011 28780312

[B2] Cervetto S. Abrevaya S. Martorell Caro M. Kozono G. Muñoz E E. Ferrari J.. (2018). Action semantics at the bottom of the brain: insights from dysplastic cerebellar gangliocytoma. Front. Psychol. 9:1194. 10.3389/fpsyg.2018.01194 30050490 PMC6052139

[B3] Chen Y. Kumfor F. Landin-Romero R. Irish M. Hodges J. R. Piguet O. (2018). Cerebellar atrophy and its contribution to cognition in frontotemporal dementias. Ann Neurol. 84, 98–109. 10.1002/ana.25271 30014499

[B4] García A. M. Abrevaya S. Kozono G. Cordero I. G. Cordoba M. Kauffman M. A.. (2017). The cerebellum and embodied semantics: evidence from a case of genetic ataxia due to STUB1 mutations. J. Med. Genet. 54, 114–124. 10.1136/jmedgenet-2016-104148 27811304

[B5] García A. M. Ibáñez A. (2016). A touch with words: Dynamic synergies between manual actions and language. Neurosci. Biobehav. Rev. 68, 59–95. 10.1016/j.neubiorev.2016.04.022 27189784

[B6] Ibáñez A. García A. M. (2018). Contextual Cognition: The Sensus Communis of a Situated Mind. Springer International Publishing.

[B7] Ibanez A. Garcia A. M. Esteves S. Yoris A. Munoz E. Reynaldo L.. (2018). Social neuroscience: undoing the schism between neurology and psychiatry. Soc. Neurosci. 13, 1–39. 10.1080/17470919.2016.1245214 27707008 PMC11177280

[B8] Ibanez A. Kuljis R. O. Matallana D. Manes F. (2014). Bridging psychiatry and neurology through social neuroscience. World Psychiatry 13, 148–149. 10.1002/wps.20125 24890065 PMC4102285

[B9] Koziol L. F. Budding D. E. Chidekel D. (2012). From movement to thought: executive function, embodied cognition, and the cerebellum. Cerebellum 11, 505–525. 10.1007/s12311-011-0321-y 22068584

[B10] Krakauer J. W. Ghazanfar A. A. Gomez-Marin A. MacIver M. A. Poeppel D. (2017). Neuroscience needs behavior: correcting a reductionist bias. Neuron 93, 480–490. 10.1016/j.neuron.2016.12.041 28182904

[B11] Roca M. Gleichgerrcht E. Ibanez A. Torralva T. Manes F. (2013). Cerebellar stroke impairs executive functions but not theory of mind. J. Neuropsychiatry Clin. Neurosci. 25, E48–E49. 10.1176/appi.neuropsych.12030057 23487232

[B12] Sokolov A. A. Miall R. C. Ivry R. B. (2017). The cerebellum: adaptive prediction for movement and cognition. Trends Cogn. Sci. 21, 313–332. 10.1016/j.tics.2017.02.005 28385461 PMC5477675

